# Importance of Apolipoprotein A-I in Multiple Sclerosis

**DOI:** 10.3389/fphar.2015.00278

**Published:** 2015-11-20

**Authors:** Lidia A. Gardner, Michael C. Levin

**Affiliations:** ^1^Research Service, VA Medical Center, Memphis, TN, USA; ^2^Department of Neurology, University of Tennessee Health Science Center, Memphis, TN, USA; ^3^Neuroscience Institute, University of Tennessee Health Science Center, Memphis, TN, USA

**Keywords:** HDL, ApoA-I, multiple sclerosis, CNS, ATP- binding cassette transporter A1, sphingosine 1 phosphate, FTY720 (fingolimod, Gilenya)

## Abstract

Jean-Martin Charcot has first described multiple sclerosis (MS) as a disease of the central nervous system (CNS) over a century ago. MS remains incurable today, and treatment options are limited to disease modifying drugs. Over the years, significant advances in understanding disease pathology have been made in autoimmune and neurodegenerative components. Despite the fact that brain is the most lipid rich organ in human body, the importance of lipid metabolism has not been extensively studied in this disorder. In MS, the CNS is under attack by a person’s own immune system. Autoantigens and autoantibodies are known to cause devastation of myelin through up regulation of T-cells and cytokines, which penetrate through the blood–brain barrier to cause inflammation and myelin destruction. The anti-inflammatory role of high-density lipoproteins (HDLs) has been implicated in a plethora of biological processes: vasodilation, immunity to infection, oxidation, inflammation, and apoptosis. However, it is not known what role HDL plays in neurological function and myelin repair in MS. Understanding of lipid metabolism in the CNS and in the periphery might unveil new therapeutic targets and explain the partial success of some existing MS therapies.

## Cholesterol Synthesis

Lipoproteins contain triacylglycerols, cholesterol esters, cholesterol and phospholipids. They exist in two main forms: high- and low-density lipoproteins (LDLs). These hydrophobic aggregates covalently bind to apoproteins to form apolipoproteins. As major components of lipoproteins, apoproteins determine their structure, metabolism, receptor interaction and function. The primary apolipoprotein of high-density lipoprotein (HDL) is apolipoprotein A-I or ApoA-I, while major component of LDL is ApoB.

In mammals peripheral cholesterol is produced in the liver and the small intestine. Cholesterol biosynthesis involves as many as 30 enzymatic steps. Cells produce cholesterol from acetyl-CoA, which is reduced to mevalonate by 3-hydroxy-3-methylglutaryl-CoA reductase (HMGCR). Through a series of reactions, mevalonate is converted to squalene and lanosterol and then to 7-dehydrocholesterol, 7-dehydrocholesterol reductase produces a cholesterol molecule. Cholesterol can be hydroxylated to 24-, 25-, and 27-hydroxycholesterol (27-OHC) by the CYP46 hydroxylase. Healthy cholesterol homeostasis is necessary for membrane formation, function of steroid hormones, vitamin D, and adequate brain function. Cholesterol is also an important myelin component and a precursor of oxysterols, steroid hormones and bile acids.

**FIGURE 1:**
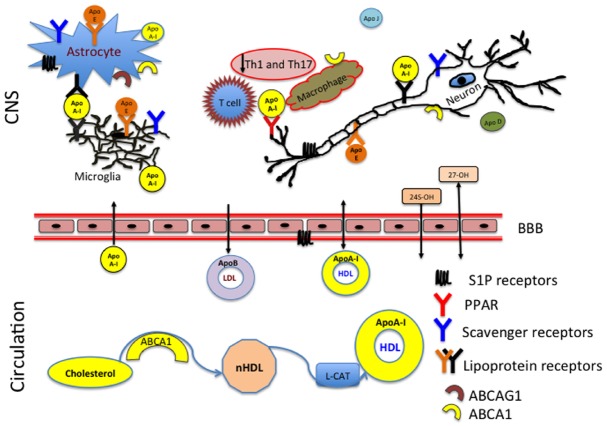
**ApoA-I reduces inflammation in the CNS by preventing contact between the T cells and macrophages.** HDL produced in the periphery has access to the CNS whereas LDL has no ability to enter the CNS from the circulation. The membrane associated ATP-binding cassette subfamily A member 1 (ABCA1) and ABCA G1 act as the primary sterol transporters for ApoA-I/HDL. HDL associated ApoA-I is then recognized by the lipoprotein receptors (LDL, SR-BI) in the CNS. Brain cholesterol homeostasis is supported by the reverse cholesterol transport and efflux of 24 (24S-OH) and 27 (27-OH) hydroxysterols through the blood–brain barrier (BBB).

Excess cholesterol is removed from tissues through a process known as reverse cholesterol transport, during which cholesterol from the periphery is returned to the liver for biliary excretion. In plasma a membrane associated ATP-binding cassette transporter A1 (ABCA1) promotes efflux of free cholesterol (FC), sphingomyelin (SM), glycerophosphocholine (PC) to ApoA-I to form nascent HDL (nHDL; Sorci-Thomas et al., 2012). This process regulates HDL levels in the periphery (Figure 1). ABCA1 also functions to contain cholesterol within lipid rafts (Vedhachalam et al., 2007; Fessler and Parks, 2011). Lipids rafts were first described as membrane microdomains consisting of both protein and lipid components (de Chaves et al., 1997).

In the central nervous system (CNS), lipid rafts are present in neurons and glia and are believed to play a role in neuronal signaling, cell adhesion and axonal guidance (de Chaves et al., 1997; Tsui-Pierchala et al., 2002). Cholesterol being at the core of a lipid raft formations, plays an important structural and functional role. In addition to cholesterol, lipid rafts contain sphingolipid enriched microdomains that regulate membrane trafficking, cell migration, and variety of signaling pathways (Simons and Ikonen, 1997).

Lipoproteins, apoproteins, and apolipoproteins can be found in circulation in free or nascent form, but lipid rafts are always associated with cellular membranes. It is unclear whether apolipoproteins require any scaffolding support of the raft for their function, but it is certain that lipid rafts need the presence of cholesterol, lipids, and proteins.

The exact role of lipid rafts in the pathogenesis of multiple sclerosis (MS) and other diseases remains to be discovered. Different brain regions have diverse cholesterol requirements and enrichment in membrane rafts (Ko et al., 2005). In addition, lipid rafts play an important role in receptor signaling. Because the brain is the most lipid rich organ in the human body it has large amounts of lipid rafts associated with brain cell membranes, lymphatic vessels and CNS vasculature (Bjorkhem and Meaney, 2004; Orth and Bellosta, 2012; Vance, 2012; Louveau et al., 2015).

## HDL Affects Sphingosine Receptors

Sphingolipids are metabolized to generate ceramide and sphingosine. Sphingosine can be phosphorylated by sphingosine kinase to generate S1P (sphingosine 1-phosphate; Scanu and Edelstein, 2008). S1P is enriched in platelets, erythrocytes, vascular endothelial cells, and plasma (Scanu and Edelstein, 2008). HDL carries biologically active S1P as part of its composition (Al-Jarallah et al., 2014). S1P receptors play a key role in lymphocyte function, which might explain the efficacy of FTY720 (fingolimod, brand name Gilenya) an oral treatment for MS. Clinical trials of FTY720 showed reduction in relapses, brain magnetic resonance imaging (MRI) lesion activity, disability progression, and brain volume loss (Kappos et al., 2006, 2010; Cohen et al., 2010). The exact mechanism of action of FTY720 in MS is not known (Cohen and Chun, 2011). However, it is presumed that FTY720 reduces infiltration of inflammatory cells through blood–brain barrier (BBB) by inhibition of lymphocyte egress from the lymph nodes. FTY720 is a synthetic sphingosine analog, which activates the S1P receptor (Mansoor and Melendez, 2008). FTY720 reduces the count of circulating lymphocytes by inducing S1P receptor internalization and degradation, which may contribute to its success in MS treatment (Pacheco et al., 2003; Spiegel and Milstien, 2003). FTY720 also affects production of IL-17 expressing T-cells and trafficking of B-cells, which has been shown to contribute to the pathogenesis of MS (Brinkmann, 2009; Mehling et al., 2010).

In addition to reducing lymphocyte quantity, S1P activates many signaling pathways including PI3K-Akt, PKC, p38MAPK, ERK1/2, and AKT-mTOR (Liu et al., 2010; Al-Jarallah et al., 2014). FTY720 reduced cholesterol toxicity in primary human macrophages, increased levels of ABCA1 and consequently efflux of endosomal cholesterol to ApoA-I, and stimulated 27-OH production (Blom et al., 2010). This and other studies highlighted the pleiotropic effect of FTY720. FTY720 might have direct CNS effects since it lowered disability progression and brain volume loss in MS patients (Cohen and Chun, 2011). S1P is produced in the CNS by astrocytes and neurons. The S1P_1_ and S1P_3_ receptors are activated in active and chronic MS lesions (Van Doorn et al., 2010). Endothelial cells and astrocytes of the BBB also express S1P receptors and FTY720 has been shown to reduce the effects of the cell death induced by inflammatory cytokines (Prager et al., 2015; Spampinato et al., 2015). Moreover, the neuroprotective effect of FTY720 could be explained by the fact that S1P receptors involved in HDL-induced inhibition of adhesion molecules [intercellular adhesion molecule 1 (ICAM-1) and vascular cell adhesion molecule 1 (VCAM-1)] expression under inflammatory conditions (Kimura et al., 2006).

## Regulation of Lipid Metabolism in the CNS

The CNS has it’s own cholesterol production and transport system. For the most part, it operates independently from peripheral cholesterol synthesis (Bjorkhem and Meaney, 2004; Orth and Bellosta, 2012; Vance, 2012). However, in diseases such as MS, the BBB is often compromised and apolipoproteins can easily carry cholesterol synthetized in the periphery to the CNS; thus altering cholesterol homeostasis behind the barrier. Brain cholesterol is synthetized by astrocytes, oligodendrocytes and neurons. Step-by-step CNS cholesterol biosynthesis is not entirely understood. However, studies have shown that enzymes responsible for cholesterol formation such as HMGCR and 7-dehydrocholesterol reductase have high expression in cortical cholinergic and hippocampal neurons (Ong et al., 2010).

The majority of the CNS cholesterol is present in unesterified form within myelin sheaths, plasma membranes of glial and neuronal cell (Bjorkhem and Meaney, 2004).

It is worthy to note that myelin lipid-protein composition is different from other cell membranes. Myelin’s dry weight is about 70% lipid and 30% protein, while other membranes have 30% lipid and 70% protein in their dry weight (Bjorkhem and Meaney, 2004). In MS, neurological symptoms are the result of loss of myelin sheaths around axons, which prevents transmission of nerve impulses. More than 95% of brain cholesterol is synthetized *de novo* from acetate (Dietschy and Turley, 2001). The HMGCR mediates the rate-limiting step of cholesterol biosynthesis. Excess cholesterol is converted into cholesterol ester by acyl CoA: cholesterol acyltransferase or to 24S-hydroxysterol (24-OH) by CYP46 expressed in neurons (Lund et al., 1999). The efflux of brain-produced cholesterol can be quantified based on the 24S-OH present in the mammalian system (Locatelli et al., 2002; Mailman et al., 2011).

In brain, oxidation of the steroid chain at position 24 is a primary mechanism of elimination of cholesterol excess (Figure 1). Outside of the brain, the oxidation occurs at position 27 by CYP27A1, expressed primarily on macrophages (Heverin et al., 2005). MS patients have decreased serum levels of both sterols: 24S-OH and 27-OH (van de Kraats et al., 2014). This suggests that disturbances in cholesterol homeostasis might relate to the neurodegeneration and disease pathology. 27-OHC produced in the periphery can penetrate through the BBB and be taken up by scavenger class B type I (SR-BI) receptors (Figure 1). The levels of 27-OHC are different in cerebrospinal fluid (CSF) of healthy adults and patients with compromised BBB (Leoni et al., 2003). SR-BI was identified as an HDL receptor in 1996 (Acton et al., 1996). SR-BI is predominantly localized to astrocytes, microglia, and macrophages (El Khoury et al., 2003; Song et al., 2015). The two most important types of receptors involved in cholesterol homeostasis are the LDL and HDL receptors. LDL receptors are highly expressed in the brain white matter and in astrocytes and their function has been extensively studied in health and disease (Kim et al., 2009; Castellano et al., 2012). The structure and property of HDL receptors continues to evolve in scientific literature (Acton et al., 1996; Webb et al., 1998; Al-Jarallah et al., 2014; Kartz et al., 2014; Chadwick et al., 2015; Song et al., 2015).

Other receptors such as peroxisome proliferator-activated receptors (PPAR) are expressed in the CNS and are involved in regulation of lipid metabolism, control of inflammation, and cholesterol transport (Bocher et al., 2002; Hulshagen et al., 2008; Chrast et al., 2011; Varga et al., 2011). PPARs form heterodimers with retinoid X receptor (RXR) and regulate inflammatory responses, myelin synthesis, neuronal cell proliferation and differentiation, energy and lipid homeostasis, and reactive oxygen species. Among the three subtypes of PPARs, PPARα, and PPARγ are present on macrophages, T cells, foam cells, and smooth muscle cells (Harris and Phipps, 2001; Yang et al., 2008). Activation of PPARα up regulates HDL production and ApoA-I expression (Mikael et al., 2006). PPARβ/δ is involved in control of brain lipid metabolism and epidermal cell proliferation (Heneka et al., 2000; Schmidt et al., 2004). The use of PPAR agonists in MS and its mouse model known as experimental autoimmune encephalomyelitis (EAE) has been explored with some positive results. For example, PPARγ ligands reduced leukocyte infiltration into the brain parenchyma and decreased both inflammation and axonal degeneration in EAE (Niino et al., 2001; Feinstein et al., 2002; Smith et al., 2004; Polak et al., 2005). PPAR antagonist GW347845 suppressed T-cell proliferation and reduced secretion of tumor necrosis factor alpha (TNFα) and interferon gamma (INFγ) in peripheral mononuclear cells (PBMCs) from MS patients (Schmidt et al., 2004). However, these effects were accompanied by reduced cell viability and induced apoptosis of inactivated lymphocytes. These studies suggest that activation of PPARs, and specifically PPARα can increase synthesis of HDL and ApoA-I to aid cholesterol biosynthesis and reverse transport.

## Major Players of Reverse Cholesterol Transport

Apolipoproteins play a major role in cholesterol recycling process—reverse cholesterol transport within the CNS and in the periphery. Production of cholesterol in the brain peaks during myelogenesis, when glial cells and neurons produce it. Mature neurons seems to loose cholesterol-producing capacity and rely instead on cholesterol delivering lipoproteins to maintain ongoing needs. Several studies indicate a role for apolipoproteins in cholesterol transfer and lipid metabolism in the CNS (Lewis et al., 2010; Takechi et al., 2010; Song et al., 2012). Among six major classes of Apolipoproteins (A, B, C, D, E, and H) only apolipoprotein E (ApoE) has been studied extensively in neurobiology. ApoE is produced by astrocytes and glial cells, and is overexpressed in human brain. ApoA-I is prevalent in the CSF but not in the brain. Abundant evidence implicates ApoE and specifically its E4 allele involvement in Alzheimer’s disease (Corder et al., 1993; Puglielli et al., 2003; Canevari and Clark, 2007). ApoA-I levels were decreased in serum, plasma and CSF of patients with Alzheimer’s disease compared to healthy controls (Kawano et al., 1995; Liu et al., 2006; Roher et al., 2009). Levels of ApoA-I decline with age, however levels below 110 mg/dL might indicate predisposition to neurodegenerative diseases. Several studies have shown that overexpression of ApoA-I prevented the development of age-related learning and memory deficits in transgenic mice (Kawano et al., 1995; Liu et al., 2006; Roher et al., 2009; Lewis et al., 2010). These studies indicate the importance of ApoA-I in the neurodegenerative diseases of the CNS.

ApoA-I is not synthetized in the brain, however it has the ability to penetrate the BBB and solicit anti-inflammatory and neuroprotective effects in the brain (Figure 1).

In addition to participating in the CNS reverse cholesterol transport, ApoA-I blocks macrophage interactions with T-cells. This results in reduction of the Th1 and Th17 associated cytokines (Figure 1). Both ABCA1 and ABCAG1 are highly expressed on astrocytes, microglia, neurons, macrophages and T cells, which indicates that there is an active cholesterol turnover between multiple components. The brain is a highly compartmentalized organ with different regions expressing different need in cholesterol synthesis and transport.

The liver X receptor (LXR) is a transcription factor expressed in the liver, macrophages, and neurons. It is noteworthy that 24S-OH is a ligand for LXRs, which activates expression of ABCA1, ApoA-I, and ApoE. Mice lacking LXRs had impaired cholesterol removal (Joseph et al., 2003). Murine deficiency in ABCA1 resulted in greater ApoA-I retention in the CNS compared to the periphery (Stukas et al., 2012). Moreover, LXR agonist GW3965 increased ApoA-I production in the brain independent from ABCA1. Thus indicating that ApoA-I may serve to integrate peripheral and CNS metabolism (Stukas et al., 2012). Overall, lipid abnormalities and cholesterol metabolism play important roles in neurological diseases (Dietschy and Turley, 2001; Bjorkhem and Meaney, 2004). Two additional apolipoproteins ApoJ and ApoD have been found in the CNS. Their function is poorly defined, beyond of proposed role as transporter proteins.

## The Role of ApoA-I in Multiple Sclerosis

ApoA-I is the most abundant component of HDL (Sorci-Thomas et al., 2012). HDL-associated ApoA-I may play a role in neuronal regeneration by acting as a constitutive anti-inflammatory factor (Hyka et al., 2001; Pfrieger, 2003; Jimenez et al., 2005). Several studies pointed out a possible protective role of ApoA-I in inflammation and autoimmunity (Panin et al., 2005; Vollbach et al., 2005; Koutsis et al., 2009; Wilhelm et al., 2009; Robciuc et al., 2010; Serban et al., 2010; Shiga et al., 2010). Differential ApoA-I expression was recognized in the CSF and serum of MS patients (Gandhi et al., 2010). In addition, ApoA-I may play a role in neuronal regeneration by acting as a constitutive anti-inflammatory factor (Burger and Dayer, 2002; Vollbach et al., 2005). However, there are major gaps in our understanding of ApoA-I regulatory mechanisms and its involvement in MS.

ApoA-I has been described as a putative clinical biomarker for interferon beta (INFβ) treatment (Gandhi et al., 2010). Study subjects, who had high levels of serum ApoA-I, responded better to INFβ therapy, a common immunomodulatory treatment for relapsing remitting MS patients. This was possibly due to the reduced inflammation associated with increased HDL levels. ApoA-I has been shown to play a role in the cognitive abilities of MS patients (Koutsis et al., 2009). Cognitive impairment is associated with a lack of APOA1 allele. Specifically, carriers of this allele performed significantly better on semantic verbal fluency and Stroop interference tests (Koutsis et al., 2009). This study evaluated 138 MS patients and 43 controls and the authors concluded that there is an association of the APOA1-75G/A promoter polymorphism with cognitive performance.

We discovered that advanced MS patients have lower plasma ApoA-I levels in comparison to patents with stable relapsing remitting disease and healthy age-matched controls (Meyers et al., 2014). Patients with primary and secondary progressive MS had the lowest levels of ApoA-I. A negative correlation between the amount of this protein and disease symptom worsening in MS was also noted. In addition, mice deficient in murine ApoA-I protein developed more severe EAE disease compared to the wild type animals with normal ApoA-I levels (Meyers et al., 2014). Our data indicate that ApoA-I levels decline with disease progression (Gardner et al., 2013; Meyers et al., 2014). Therefore, preventing ApoA-I levels from decreasing might prove beneficial for MS patients.

Another group assessed serum lipid profiles of 492 MS patients for associations with disability and MRI outcomes (Weinstock-Guttman et al., 2011). The authors reported that higher HDL was associated with lower levels of acute inflammation and worsening in the expanded disability status scale (EDSS). High EDSS was associated with higher baseline LDL and total cholesterol. A prospective population–based cohort study found an association between adverse lipid profile and high levels of MS disability and disease progression (Tettey et al., 2014). The authors shown that total cholesterol, ApoB and ApoB/ApoA-I ratio were independently associated with higher EDSS.

Given the fact that ApoA-I is expressed at high levels in spinal fluid, and perturbations in lipid metabolism negatively affect myelin, factors that control ApoA-I production and turnover should receive special consideration (Hulshagen et al., 2008; Chrast et al., 2011; Levin et al., 2014). Levels of ApoA-I could be raised with statins, however use of statin medication in MS patients delivered conflicting results (Vollmer et al., 2004; Lock, 2008; Maier et al., 2009; Markovic-Plese et al., 2009; Chataway et al., 2012).

A pilot study of 30 MS patients demonstrated a significant decrease in the number and volume of contrast enhancing lesions with 80 mg of simvastatin treatment (Vollmer et al., 2004). A large safety and efficacy of natalizumab in combination with INFβ-1A in patents with relapsing remitting multiple sclerosis (SENTINEL) study did not indicate any effect of statins on relapse rate, disability progression or the number of contrast enhancing lesions (Rudick et al., 2009). Simvastatin treatment combined with INFβ did not provide benefit for MS patients (Sorensen et al., 2011). A recent double blind, placebo-controlled MS-STAT clinical trial has shown that high dose simvastatin attenuates brain atrophy, the main reason of which is believed to be a decreased neuroaxonal loss (Chataway et al., 2014). This study, recruited MS patients with secondary progressive disease, who did not receive other disease modifying drugs. Reduction in total cholesterol levels correlated with reduction in brain atrophy in this trial. The authors did not directly measure ApoA-I levels in MS patients, however because of the known effects of simvastatin on cholesterol metabolism (Matthan et al., 2003), the data strongly suggest that HDL and Apo A-I levels were higher in simvastatin group, where cholesterol was reduced from 5.5 to 4.1 mmol/L and brain atrophy was reduced by 43% (Chataway et al., 2014).

Low ApoA-I presence in progressive patients’ plasma as we discovered in our study (Meyers et al., 2014) could explain success of high dose simvastatin treatment in this large placebo controlled phase 2 trial (Chataway et al., 2014). The controversial results of statin use in MS could be partially elucidated by the fact that statins increase reactive oxygen species, elevate lipid peroxidation and induce oxidative DNA damage in human peripheral blood lymphocytes (Gajski et al., 2008; Qi et al., 2013). Increased lipid peroxidation is associated with disease exacerbation periods and lesion pathogenesis in MS patients (Toshniwal and Zarling, 1992; Levin et al., 2013). Therefore different type of drugs, which stimulate ApoA-I production, might prove beneficial for progressive MS patients.

## Conclusion

Apolipoproteins are important players in cholesterol homeostasis. ApoA-I acting as a major HDL component is involved in both HDL biosynthesis and transport. In CNS diseases with compromised BBB, healthy cholesterol turnover becomes extremely important for neuronal homeostasis and regeneration. In as much as the S1P receptor agonist fingolimod, Apo-I’s function is not completely understood in MS. However, data suggests a positive neuroprotective effect of this apolipoprotein on the immune and the CNSs. Reduction in ApoA-I levels has not been shown to cause MS. However for progressive patients, maintaining normal levels of ApoA-I might result in better neuronal health. Agents designed to improve ApoA-I production should be considered for therapeutic purposes. For example, PPAR agonists are one class of medications that regulate ApoA-I and subsequently HDL synthesis. Therefore, studies aimed at compounds responsible for ApoA-I expression during periods of inflammation could provide important information about the mechanisms of HDL regulation and its role in MS pathogenesis.

### Conflict of Interest Statement

Dr. Gardner became an employee of Novartis after initial submission of this manuscript. The authors declare that the research was conducted in the absence of any commercial or financial relationships that could be construed as a potential conflict of interest.
